# Microarray Analysis Uncovers a Role for Tip60 in Nervous System Function and General Metabolism

**DOI:** 10.1371/journal.pone.0018412

**Published:** 2011-04-11

**Authors:** Meridith Lorbeck, Keerthy Pirooznia, Jessica Sarthi, Xianmin Zhu, Felice Elefant

**Affiliations:** Department of Biology, Drexel University, Philadelphia, Pennsylvania, United States of America; City of Hope National Medical Center and Beckman Research Institute, United States of America

## Abstract

**Background:**

Tip60 is a key histone acetyltransferase (HAT) enzyme that plays a central role in diverse biological processes critical for general cell function; however, the chromatin-mediated cell-type specific developmental pathways that are dependent exclusively upon the HAT activity of Tip60 remain to be explored.

**Methods and Findings:**

Here, we investigate the role of Tip60 HAT activity in transcriptional control during multicellular development *in vivo* by examining genome-wide changes in gene expression in a *Drosophila* model system specifically depleted for endogenous dTip60 HAT function.

**Conclusions:**

We show that amino acid residue E431 in the catalytic HAT domain of dTip60 is critical for the acetylation of endogenous histone H4 in our fly model *in vivo*, and demonstrate that dTip60 HAT activity is essential for multicellular development. Moreover, our results uncover a novel role for Tip60 HAT activity in controlling neuronal specific gene expression profiles essential for nervous system function as well as a central regulatory role for Tip60 HAT function in general metabolism.

## Introduction

The Tat-interactive protein-60 KDa (Tip60) is a member of the MYST histone acetyltransferase (HAT) super family [1], first identified based on its interaction with the human immunodeficiency virus, type 1-encoded transactivator protein Tat [2]. Tip60 has been reported to play essential roles in a wide variety of cellular processes based upon the different protein complexes it is transiently associated with. The majority of cellular Tip60 protein purifies as part of a stable and conserved multimeric Tip60 protein complex containing at least 18 subunits [3]. Importantly, this Tip60 complex is evolutionarily conserved from *Saccharomyces cerevisiae* to *Drosophila* to humans [4], [5], [6], making it amenable for functional characterization using multiple model systems. Such studies have revealed that a number of the Tip60 interacting protein partners within the Tip60 complex are specifically required for the diverse and general cellular processes that Tip60 regulates, including cell cycle and checkpoint control, apoptosis, and DNA damage repair [3], [7], [8], [9]. Tip60 can also be recruited to the promoters of specific target genes *via* its transient interaction with a variety of different transcription factors to either activate or repress gene expression. Activation requires the epigenetic HAT function of Tip60, which acts to acetylate the nucleosomal histones of target genes [10]. Acetylation promotes chromatin disruption that in turn, facilitates additional factor binding and transcriptional activation [11], [12], [13], [14]. Repression is thought to be independent of Tip60 HAT activity, and may result from its interaction with transcriptional silencers and histone deacetylases [15]. Tip60 HAT activity also functions to directly acetylate certain transcription factors (TFs), which serves to activate or repress their respective gene regulatory functions [15].

Experiments coupling chromatin immunoprecipitation (ChIP) with hybridization of oligonucleotide arrays in *Saccharomyces cerevisiae* demonstrate that Esa1, the yeast Tip60 homolog, is recruited to the promoters of virtually all active protein-coding genes [16]. However, a similar role for Tip60 in general gene activation remains to be determined during metazoan development where robust and preferential Tip60 protein localization profiles in the developing myocardium and brain in chicken and mouse [17] have been observed and Tip60 cell type specific activity and preferential brain and heart tissue-specific expression patterns have been reported [17], [18]. Indeed, studies in mammalian cells have revealed that Tip60 transiently associates with a growing list of specific transcription factors where it acts as a coactivator or corepressor for certain target genes [8], [19], [20], [21], [22], [23]. Notably, TIP60 was recently identified as one of the six ‘hub’ genes uncovered in a large-scale genetic interaction screen in *C. elegans*, so characterized by their ability to interact with multiple other genes and with all of the developmental signaling pathways screened in the study [24]. Moreover, RNAi depletion studies of Tip60 in an embryonic stem cell (ESC) line demonstrated that Tip60 represses a large number of developmental genes essential for ESC differentiation, and as such, identified Tip60 as a regulator of ESC identity [25]. However, despite the undisputed central role that Tip60 plays in the regulation of general developmental gene control, the question of whether the epigenetic HAT activity of Tip60 is required for differential tissue-specific gene expression profiles essential for organismal development remains to be explored.

Here, we investigate the role of Tip60 HAT function in transcriptional control during multicellular development *in vivo* by examining genome-wide changes in gene expression in a *Drosophila* model system specifically depleted for endogenous dTip60 HAT function. Our results support a critical role for dTip60 catalytic HAT residue E431 in the acetylation of endogenous histone H4 in our fly model, *in vivo* and demonstrate that dTip60 HAT activity is essential for multicellular development. Moreover, our results uncover a novel role for Tip60 HAT activity in controlling neuronal specific gene expression profiles essential for nervous system function as well as a central regulatory role for Tip60 HAT function in general metabolism.

## Results

### Expression of mutant HAT defective dTIP60^E431Q^ produces a dominant negative lethal effect during *Drosophila* multicellular development

**Figure 1:**
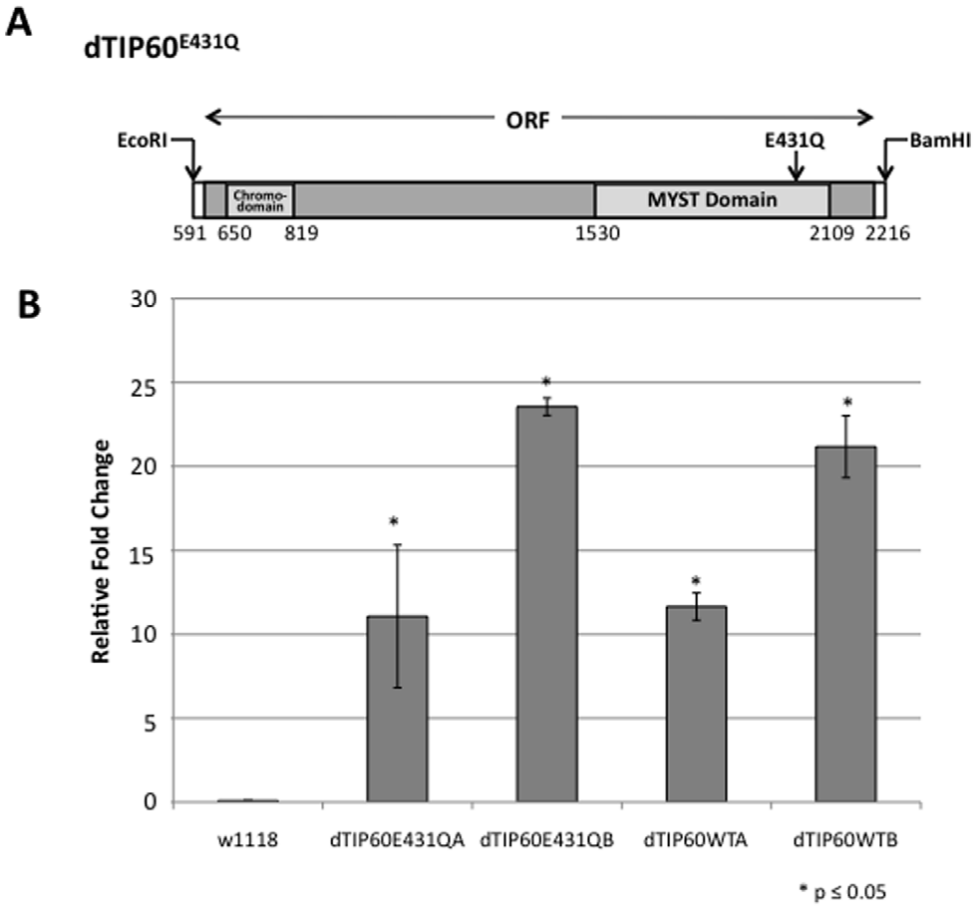
Generation and characterization of transgenic dTip60^E431Q^ and dTip60^WT^ flies. (**A**) Schematic of the dTIP60 open reading frame. Shown is the location of the conserved regions encoding for the N-terminal chromo domain and the C-terminal MYST functional domain. An arrow denotes the position site of amino acid substitution E431Q. (**B**) Exogenous expression levels of dTip60^E431Q^ and dTip60^WT^ in independent fly lines. Shown is a histogram depicting qPCR analysis of exogenous levels of dTip60 in staged three day old second instar larvae progeny resulting from a cross between ubiquitous GAL4 drive 337 and either dTip60^E431Q^ (lines A and B), dTip60^WT^ (lines A and B) or control w^1118^ flies. To quantify the amount of exogenous expressed dTip60^E431Q^ or dTip60^WT^, primers were designed to amplify a 97bp non-conserved region of dTip60 (present in both endogenous and exogenous dTip60) and a 105bp non-conserved region within the 5′UTR of dTip60, (present only in endogenous dTip60). For each transgenic line, RNA was extracted and qPCR performed using Power Sybr Green. The cycle threshold (CT) was determined for each primer pair and normalized to the CT value of RP49 (ribosomal protein internal loading control) to account for differences between samples. The relative fold change in mRNA expression levels between exogenous and endogenous dTip60 was measured using the comparative Ct method with Rp49 as the internal control, and these results are summarized in the histogram. Asterisks (*) indicate significant fold change between the respective genotype and control flies with values of p≤0.05; n = 3. Error bars represent standard error of the mean.

We previously identified and cloned the human homologue of TIP60 in *Drosophila*, referred to as Dmel\TIP60 [6], [26] or dTip60 [5] and demonstrated by GAL4 targeted RNAi knockdown technology [27] that ubiquitous reduction of endogenous Dmel\TIP60/RNAi in the fly results in lethality [6]. These results support an essential role for the dTip60 protein in multicellular development. To extend these studies, and investigate the epigenetic dependency of fly development on Tip60 HAT function, we set out to create a fly line producing GAL4 inducible dominant negative acting dTip60 proteins specifically defective in their catalytic HAT activity. These flies could serve as an experimental tool to identify those cellular processes and genes directly reliant upon the epigenetic HAT function of Tip60 (dTip60^E431Q^ lines) rather than those that may also require additional Tip60 protein function (dTip60^RNAi^), as well as potentially limit off- target gene disruption that may occur when using RNAi knockdown. Additionally, unlike our dTip60^RNAi^ knockdown construct, expression of dTIP60^E431Q^ presumably interferes with the dTip60 protein already produced by maternal dTip60 RNA in the embryo, thus more effectively interfering with dTip60 protein function *in vivo* in the fly. The mutant dTip60 construct was created by introducing a specific amino acid substitution E431Q into the conserved enzymatic HAT domain of dTIP60 that corresponds to mutation E338Q in the yeast Tip60 homolog Esa1 [28] (Figure 1A). Importantly, the Esa1(E338Q) mutant protein has been shown to retain proper protein folding, exhibit substantially reduced HAT activity, and exhibit a dominant negative effect in yeast cells [28]. Flies were transformed with dTIP60^E431Q^ within a GAL4 inducible pUAST construct, and independently derived transgenic fly lines were chosen for initial characterization. The insertions were homozygous viable, and did not cause any observable mutant phenotypes in the absence of GAL4 induction.

The amino acid residue E338 in the catalytic HAT domain of the yeast Esa1 protein is thought to be crucial for catalysis, however a conserved function for this residue in the Tip60 protein of multicellular organisms was unknown. To determine whether dTip60^E431Q^ would cause a dominant negative effect during fly development, we induced expression of either mutant dTip60^E431Q^ (independent lines A–C) or exogenous wild-type dTip60 designated dTip60^WT^ (independent lines A–C) at 25°C using the GAL4 driver 337 [29]. This driver produces robust and ubiquitous GAL4 production beginning during late embryonic development and continuing into adulthood. The w^1118^ fly line crossed to 337-GAL4 served as a control. We found that control flies as well as two independent fly lines each expressing wild-type dTip60^WT^ all exhibited normal phenotypes. However, induction of dTip60^E431Q^ for independent lines A–C reduced fly viability to 0% (Table 1). The developmental stage when lethality occurred varied between individual fly lines, with the majority of lethality occurring during the late pupal stage for line A, and during the late second instar larvae stages for lines B and C. Such variation in developmental stages of lethality may be due to position effect variegation on expression levels due to random transgene insertion within the genome (Figure 1B). Similar results were obtained using the ubiquitous actin driver Act5c for four independent dTip60 ^E431Q^ lines (lines A–D; Table S1), confirming the dominant negative lethal effects observed. Taken together, these results demonstrate that production of dTip60^E431Q^ produces dominant negative lethal effects during fly development, and that the HAT catalytic activity, which is dependent on the presence of E431 in the catalytic site, is essential for dTIP60 function in multicellular development.

**Table 1:** Ubiquitous expression of dTIP60 in independent fly lines produces a dominant negative lethal effect that can be rescued by an additional copy of wild-type dTIP60.

Fly Lines x 337-GAL4	
Test Cross Fly Lines^a^	Number of Surviving Adult Flies^c^
w^1118^	99±3
dTIP60^E431Q^A	0±0*
dTIP60^E431Q^B	0±0*
dTIP60^E431Q^C	0±0*
dTIP60^WT^A	120±28*
dTIP60^WT^B	107±18
dTIP60^WT^C	116±14
**Rescue Cross Fly Lines** b	
dTIP60^Rescue^A	65±23*
dTIP60^Rescue^B	97±9
dTIP60^Rescue^C	110±10
dTIP60^Rescue^D	56±7*
dTip60^UAS titration control^	0±0

*p≤0.05.

a Test Cross Fly Lines. Ten flies homozygous for either dTip60^E431Q^ or dTip60^WT^ P-element insertions, or control w^1118^, were mated to seven flies homozygous for the ubiquitous 337-GAL4 driver. For independently derived fly lines dTip60^E431^ A, B and C the P-element insertions are located on chromosome 3, and for independently derived fly lines dTip60^WT^ A, and C the P-element insertions are located on chromosome 2.

b Rescue Cross Fly Lines. Four independent rescue lines were generated, each homozygous for dTip60^WT^ (line A or B) on the second chromosome and dTip60^E431Q^ (line A or B) on the third chromosome. Rescue lines are designated as follows: line A is dTip60^E431Q^ B/dTip60^WT^A, line B is dTip60^E431Q^ B/dTip60^WT^B, line C is dTip60^E431Q^ A/dTip60^WT^A, line D is dTip60^E431Q^ A/dTip60^WT^B. UAS titration control is dTip60^E431^B/UAS-GFP which is homozygous for UAS-dTip60^E431^ line B on the third chromosome and UAS-GFP (UAS driven green fluorescent protein) on the second chromosome. Ten homozygous flies for each of the independent rescue fly lines were crossed to seven flies homozygous for the ubiquitous 337-GAL4 driver.

c Number of Surviving Adult Flies. Adult progeny were counted over an eight day period and the total number of surviving flies was scored. Both dTip60^E431Q^ lines reduced viability to 0% that of w^1118^ control flies. For dTIP60^WT^ line A, there was a significant increase in survivorship when compared to control w^1118^ flies. All four rescue lines showed significant rescue of the observed lethal phenotype, with rescue lines A and D exhibiting greater than 50% rescue and rescue lines B and C exhibiting 100% rescue. The UAS titration control dTip60^E431^B/UAS-GFP showed no rescue, indicating that rescue is dependent upon additional dTip60^WT^ protein, and not potential GAL4 titration due to the additional UAS construct. The results are reported as mean ± SD (n = 3); * p≤0.05.

**Figure 2:**
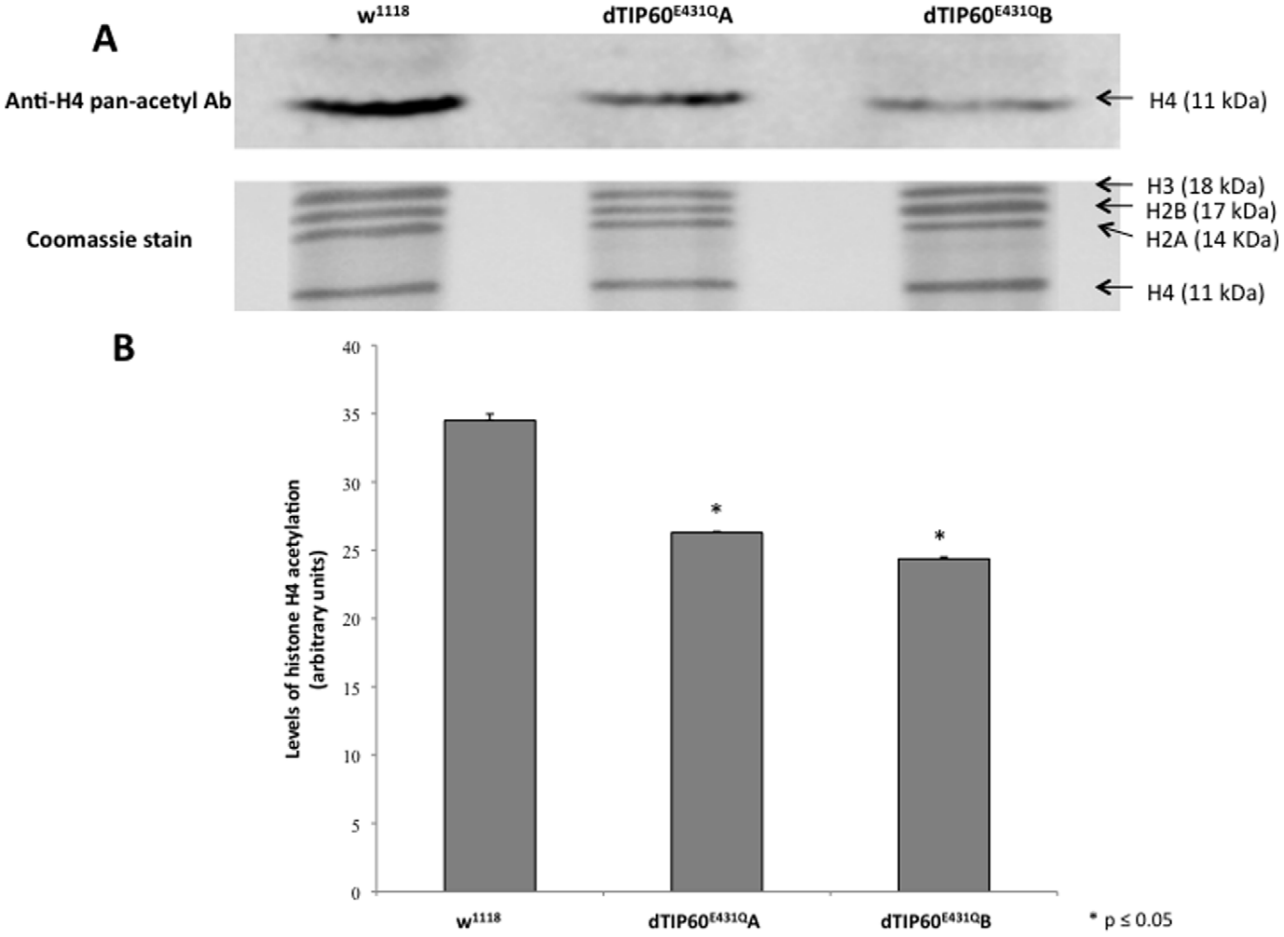
Expression of dTip60^E431Q^ in flies significantly depletes endogenous levels of histone H4 acetylation *in vivo*. (**A**) Equal amounts of core histones isolated from 50 three day old staged second instar larvae for each genotype crossed to GAL4 line 337 were resolved by 18% polyacrylamide gel electrophoresis, Western-blotted, and immunostained with antibodies that recognize four acetylated lysine residues (K5, K8, K12 and K16) of histone H4. Coomassie staining of core histone proteins were used to ensure equal loading of the samples [30], [31]. (**B**) Western blot signals were quantitated using Fluorchem imager (Alpha Innotech) and the results are summarized in the histogram depicting arbitrary units of endogenous histone H4 acetylation for each of the three genotypes analyzed. To ensure signal was in the linear range, Alpha Ease FC software (Alpha Innotech, San Leandro, CA) was used according to the manufacturer's instructions to select exposure times such that there was no saturation detected. Values indicated are the mean of three independent biological replicates. Asterisks (*) indicate significant fold change in acetylation in relation to control w^1118^ flies where p<0.05. Error bar depicts standard error of the mean; n = 3.

The mutant dTip60^E431Q^ protein theoretically produces a dominant negative effect in the fly by outcompeting endogenous wild-type dTip60 for binding to the dTip60 complex when over-expressed. To determine whether the severity of the dominant negative effect correlates with dTip60^E431Q^ expression levels and thereby its ability to outcompete the wild type, we used qPCR to compare the exogenous levels of dTip60^E431Q^ transgene expression between fly lines A and B, as they exemplified the greatest and least severe dominant negative phenotypes, respectively using GAL4 ubiquitous driver 337 for induction (Table 1). Determination of transgene induced exogenous dTip60^E431Q^ or dTip60^WT^ for each line was accomplished by amplifying total dTip60 mRNA using primers designed to a non-conserved region within both the endogenous and exogenous transgene induced dTip60, and calculating the relative fold change in mRNA expression levels in comparison to endogenous dTip60 mRNA levels using primers designed specifically to the endogenous 5′UTR dTip60 region that is lacking in the exogenous transgene induced dTip60. The relative fold change in mRNA expression levels between exogenous and endogenous dTip60 was measured using the comparative Ct method with RP49 as the internal control. All samples analyzed were early second instar larvae, as this is the stage directly before dTip60^E431Q^ induced lethality occurs. We found that both lines expressed exogenous dTip60^E431Q^, with line B expressing almost twice the level of dTip60^E431Q^ than line A, possibly suggesting that the more severe dominant negative effect of line B may be due to the greater level of dTip60^E431Q^ it produces (Figure 1B). Of note, although comparably robust levels of exogenous wild-type dTip60 were observed for dTip60^WT^ fly lines A and B, unlike dTip60^E431Q^ fly lines, both dTip60^WT^ fly lines exhibited normal phenotypes. To determine whether induction of HAT-defective dTIP60^E431Q^ leads to depletion of endogenous histone H4 acetylation levels, *in vivo* we carried out Western analysis [30], [31] on equal amounts of endogenous histone proteins purified from each of the second instar larval samples using antibodies to pan-acetylated histone H4, which is the preferential histone substrate of Tip60. Our results reveal that endogenous levels of acetylated histone H4 are significantly depleted in both independent fly lines dTip60 A and B when compared to control samples (Figure 2 A and B). Taken together, these results demonstrate that the dominant negative effect is dependent upon the level of mutant dTip60^E431Q^ produced, and that the amino acid E431 in the catalytic HAT domain of dTip60 is critical for acetylating endogenous histone H4 *in vivo*.

To confirm that the lethal effects we observed were specifically caused by defective dTip60^E431Q^ function, we assessed whether additional levels of GAL4 induced wild-type dTIP60 would rescue dTip60^E431Q^ induced lethality. Four independent fly strains were produced that were homozygous for different combinations of both the strongest or weakest expressing dTip60^E431Q^ transgene and the strongest or weakest expressing dTIP60^WT^ transgene in addition to the endogenous dTIP60 gene on the X chromosome. These fly lines were designated as independent rescue lines dTip60^Rescue^ A, B, C, or D (Table 1). Each of these fly lines were crossed to the ubiquitous GAL4 driver 337 and the viability of the progeny was scored (Table 1). The results revealed that in this genetic background, when additional wild-type dTIP60 transgene (dTip60^WT^) expression was induced by GAL4 in flies also expressing the GAL4 induced dTIP60^E431Q^ construct, a significant number of flies were rescued with 100% rescue for two of the four rescue lines (Table 1). Similar rescue results by dTip60^WT^ were obtained for dTip60^RNAi^ induced lethality [5] as well as using a second ubiquitous driver Act5c (Table S1). These results demonstrate that dTIP60^E431Q^ induced lethality is specifically caused by over-expression of the mutant protein, as this effect can be rescued by additional expression of wild-type dTip60. These findings as a whole demonstrate that the HAT activity of dTip60 is essential for *Drosophila* multicellular development, and support our system as a valuable *in vivo* model for investigating the epigenetic based dependency of developmental processes on Tip60 HAT function.

### dTip60 HAT activity is required for the transcriptional regulation of genes involved in a diverse array of metabolic and general cellular processes

To gain insight into the role of Tip60 HAT function in transcriptional control during multicellular development, we used microarray analysis to examine changes in gene expression in response to ubiquitous induction of either dTip60^E431Q^ or dTip60^WT^ in the fly. Our highest expressing transgenic fly lines dTip60^E431Q^ line B, dTip60^WT^ line B, and w^1118^ control flies were each crossed to the ubiquitous GAL4 driver 337. As induction of dTip60^E431Q^ with the 337-GAL4 driver results in lethality during late second instar larval stage, RNA samples were isolated from 35 three-day-old pooled larvae collected prior to lethality, to enhance our opportunity to detect Tip60 related cellular changes and ensure that such changes were not linked to tissue necrosis. Microarray analysis was carried out in duplicate on these pooled biological replicate samples using the Affymetrix *Drosophila* Genome 2.0 Array. A correlation matrix generated using dCHIP software demonstrated that the correlation coefficients calculated for duplicate samples for each of the three genotypes analyzed showed significant agreement, indicating high reproducibility of the gene expression data we present in this study. Genes selected for misregulation were identified as those with a fold change of greater than 2 or less than −2 (p≤0.05) between the w^1118^ control and dTip60^E431Q^ or dTip60^WT^ fly lines after normalization and standardization using dChip programs.

**Figure 3:**
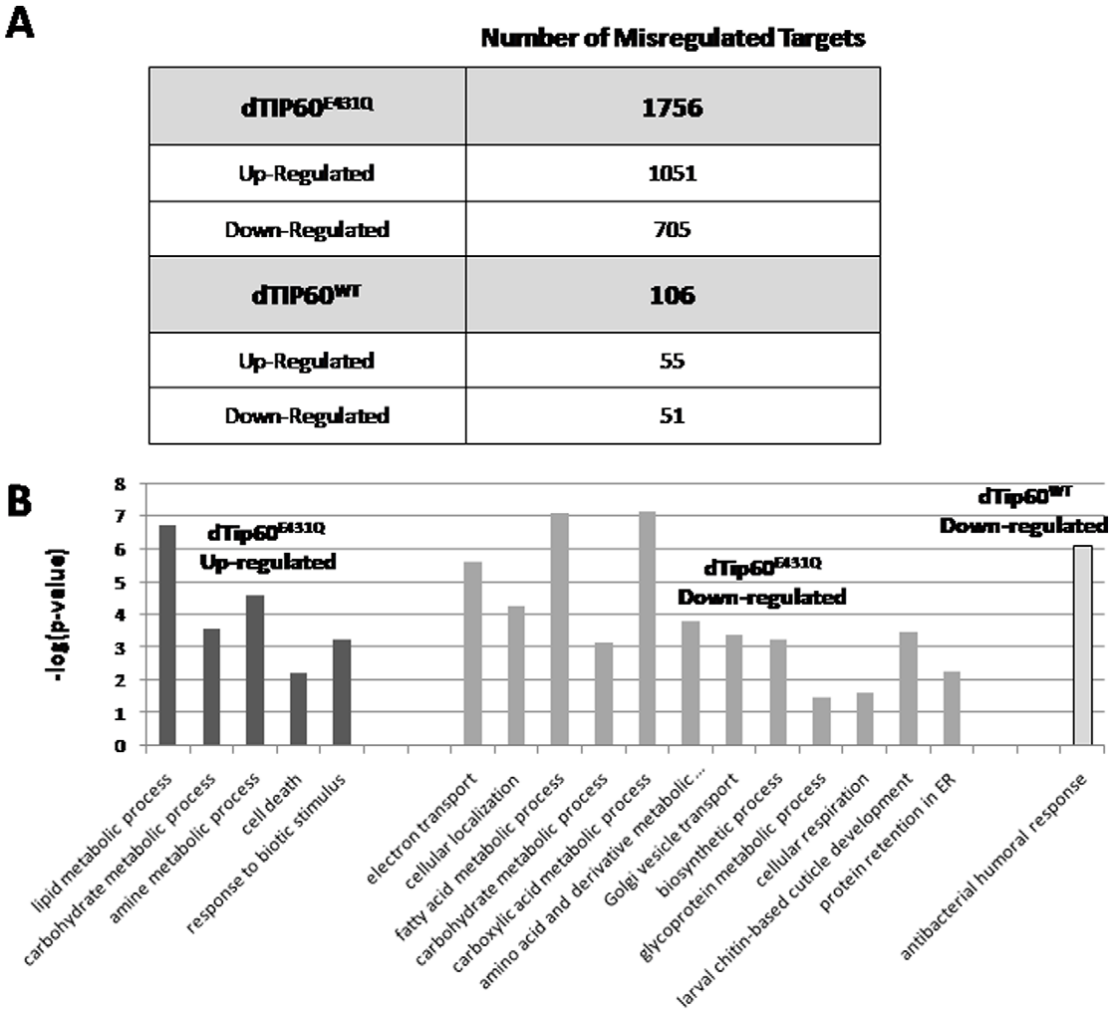
Microarray analysis reveals a central role for Tip60 in the transcriptional control of genes linked to diverse metabolic and general cellular processes. (**A**) Total number of significantly misregulated genes in response to dTip60^E431Q^ or dTip60^WT^. The dCHIP t-test function was used to identify genes whose expression differed significantly (p<0.05) and these genes were then filtered to select for those that showed a twofold or greater change and a 90% confidence bound of fold change. (**B**) Significantly enriched gene ontology (GO) groups representing dTip60^E431Q^ and dTip60^WT^ misregulated genes. Genes were annotated and biological processes were analyzed using the Database for Annotation, Visualization, and Integrated Discovery (DAVID). Significance of overrepresentation of Gene Ontology (GO) terms was determined (p<0.05). Enrichment score (y-axis) is reported as the minus log transformation on the geometric mean of p-values from the enriched annotation terms associating with one or more of the gene group members. The genes up-regulated in response to dTip60^E431Q^ clustered into 5 significantly enriched groups, and down-regulated genes clustered into 12 significantly enrichment groups, with 8 of these groups enriched for metabolic processes. Genes misregulated in response to dTip60^WT^ grouped to one significantly enriched cluster.

We identified a total of 1756 genes that were significantly misregulated in response to dTip60^E431Q^ induction, with 1051 genes up-regulated, 705 genes down-regulated (Figure 3A). In contrast, only 106 genes were identified that were significantly misregulated in response to dTip60^WT^ induction in comparison to control samples, with 55 genes up-regulated and 51 down-regulated (Figure 3A), and 22 genes that were misregulated in response to both dTip60^E431Q^ and dTip60^WT^ (Table S2). This minimal number of genes misregulated by dTip60^WT^ was not surprising as induction of dTip60^WT^ in the fly leads to no observable phenotypic effects (Table 1). Importantly, the comparable levels of expression that we observed for ubiquitous induction of exogenous dTip60^E431Q^ and dTip60^WT^ in the fly (Figure 1B) argue that the significantly larger number of misregulated genes we identify in response to dTip60^E431Q^ expression are specifically due to consequences of the amino acid substitution in the HAT domain of dTip60, rather than simply an artifact caused by over-expression of the transgene itself.

To identify biological processes that were significantly affected as a result of gene misregulation, we utilized the DAVID Functional Annotational Clustering tool [32], [33] to group the misregulated genes into clusters by their gene ontology (GO) based on biological process. The genes up-regulated in response to dTip60^E431Q^ clustered into 5 significantly enriched groups (p<0.05) that represent lipid metabolism, carbohydrate metabolism, amine metabolism, cell death, and response to biotic stimulus (immune) processes (Figure 3B). Down-regulated genes clustered into 12 significant (p<0.05) enrichment groups representing electron transport, cellular localization (protein), fatty acid metabolism, carbohydrate metabolism, amino acid metabolism, Golgi vesicle transport, biosynthetic processes (translation), glycoprotein metabolism, cellular respiration, larval chitin-based cuticle development, and protein retention in ER (Figure 3B). Interestingly, although there were more genes up-regulated in response to dTip60^E431Q^, only 5 significantly enriched gene ontology clusters were identified as compared to the 12 identified for up-regulated genes, indicating that the upregulated genes are not as enriched in specific biological processes as the downregulated ones. Of note, up-regulated genes in response to dTip60^WT^ did not group into any significant clusters and down-regulated genes grouped into only one significant cluster that related to bacterium responses, consistent with the lack of phenotypic effects resulting from dTip60^WT^ over-expression in the fly. Taken together, our microarray results support a role for dTip60 in the control of target genes involved in a diverse array of metabolic and general cellular processes.

### dTip60 HAT activity is required for neuronal gene expression profiles and is essential for nervous system function

**Figure 4:**
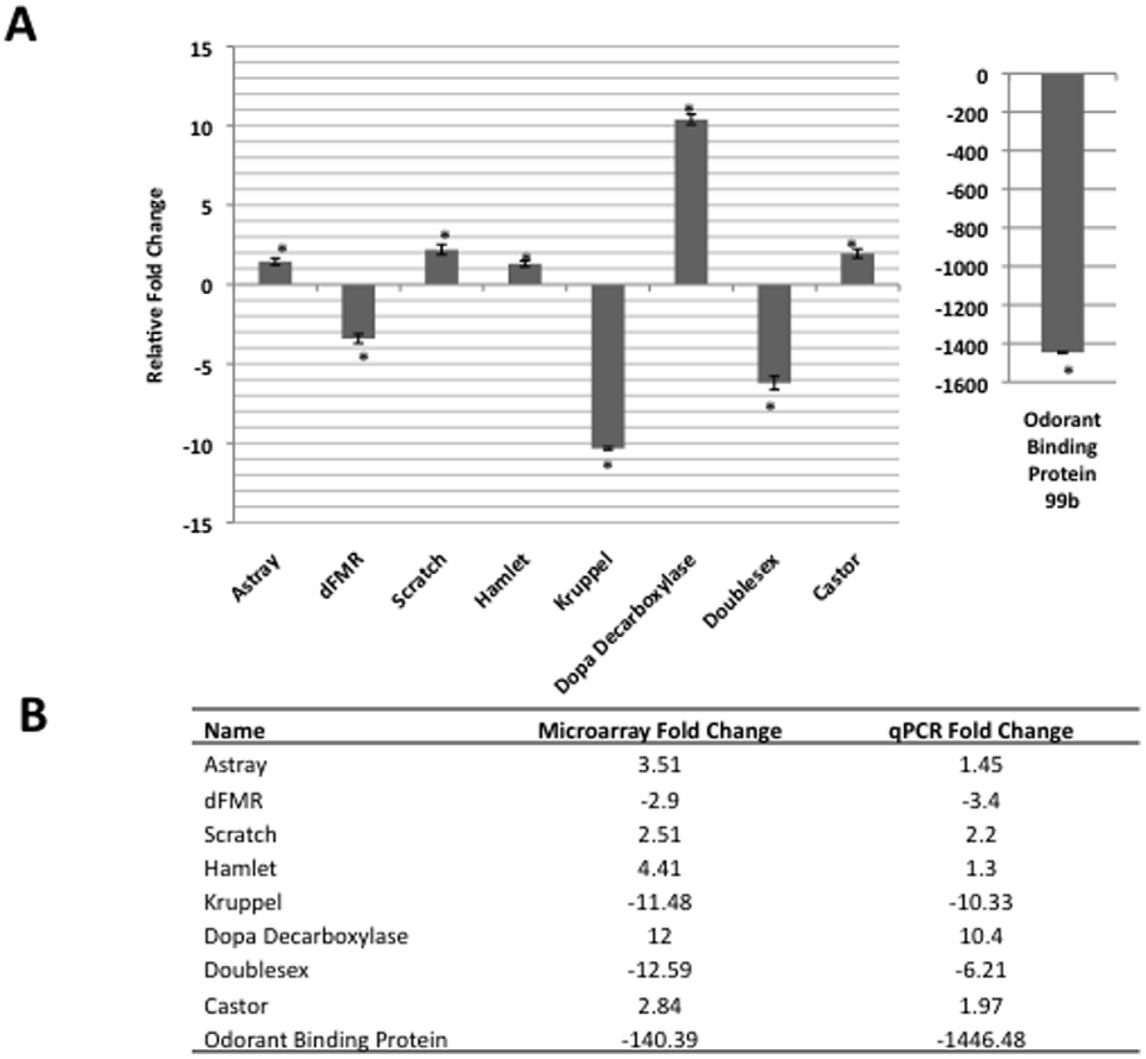
qRT-PCR validation of selected neuronal target genes identified by microarray analysis. Shown is a histogram depicting qPCR analysis of the expression of selected neuronal target genes identified by microarray using aliquots of cDNA pools prepared for microarray analysis. The relative fold change in mRNA expression levels were measured using the comparative Ct method with RP49 as the internal control gene. Asterisk (*) indicates significant fold change where * is p≤0.05, ** is p≤0.0005 and *** p≤0.000008. Error bars represent standard deviation and are reported as ± standard error of the mean, (n = 3).

The majority of significantly misregulated genes affected by depletion of Tip60 HAT activity grouped to 17 significantly enriched main and general categories representing general metabolic and cellular processes. However, as the microarray analysis was carried out on a mixed population of cells extracted from developing, whole second instar larvae, these *in vivo* samples gave us the opportunity to investigate whether depletion of Tip60 HAT activity also affected genes linked to tissue and cell type specific biological processes as well as the general cellular processes we had identified. Further analysis by DAVID of the genes that did not group to the 17 main clusters but were still significantly misregulated (p≤0.05), revealed additional clusters enriched for cell cycle control regulators, genes involved in general cell development and intriguingly, genes enriched for 17 categories all relating to neuronal function and development, with 7 clusters linked to down-regulated genes and 10 clusters linked to up-regulated genes (Table 2). The neuronal processes identified were diverse, with functions linked to behavior, learning and memory, as well as sensory, neurogenesis and general neuronal system function. Of note, aside from one muscle-development related cluster, these neuronal categories were the only tissue-specific related clusters identified in our analysis. To validate the microarray results, we carried out qRT-PCR analysis on 11 genes encoding proteins with known function. The up-regulated and down-regulated genes selected for this analysis represented a wide range of neuronal functions including neuronal cell type differentiation, transmission of nerve impulses, locomotion and behavior, learning and memory, as well as sensory processes including sight and olfactory behavior. A comparison of the microarray data and qRT-PCR of selected targets showed good correlation (Figure 4 A and B), indicating the reliability of our microarray data as well as supporting a role for dTip60 in the regulation of a wide variety of genes required for neuronal development and function.

**Table 2:** Gene ontology clusters significantly misregulated in response to dTIP60^E431Q^.

Biological Process^a^	Number of Targets	Biological Process^a^	Number of Targets
**Up-regulated**		**Down-regulated**	
Response to Stress	9	Protein Processing	5
Growth	14	Mitochondrion Organization and Biogenesis	7
Lipid Biosynthetic Process	11	Metabolic Process (Protein Metabolic Process)	257
Response to External Stimulus^b^	11	DNA Metabolic Process	12
Behavior (Chemosensory, Learning and Memory)^b^	23	Aromatic Compound Metabolic Process	9
Pigment Biosynthetic Process	7	Heterocycle Metabolic Process	9
Membrane Lipid Metabolic Process	9	Response to Abiotic Stimulus	9
Positive Regulation of Growth	4	Vesicle-Mediated Transport	24
Cell Adhesion	18	Secondary Metabolic Process (Pigment Biosynthetic Process)	6
Cellular Process (Protein Metabolic Process)	328	Behavior (Courtship Behavior)^b^	11
Sensory Perception^b^	18	Regulation of Gene Expression	14
Amino Acid Derivative Metabolic Process	5	Cell Differentiation (Cell Death)	26
Apoptosis	11	Aging	4
Cellular Catabolic Process	14	Oogenesis	14
Amine Transport (Amino Acid Catabolic Process)	14	Amino Acid Biosynthetic Process	3
Amino Acid Transport	4	Cellular Homeostasis	5
Regulation of Hydrolase Activity	5	Salivary Gland Development	6
Circadian Rhythm^b^	4	Transcription (Reproductive Process)	9
Reproductive Process (Reproductive Developmental Process)	11	Nervous System Development (Apoptosis)^b^	10
Reproductive Process (Mating Behavior)^b^	11	Response to Abiotic Stimulus	9
Embryonic Development	23	Sensory Perception^b^	10
Aging	5	Response to Biotic Stimulus (Immune System Process)	5
Muscle Development^c^	8	Embryonic Development	11
Neurological System Process^b^	36	Cellular Developmental Process (Cell Differentiation, Gamete Generation)	26
Regulation of Programmed Cell Death	8	RNA Processing	7
Cellular Homeostasis	7	Cell Cycle	15
Vesicle-Mediated Transport	22	Protein Modification Process	35
Locomotory Behavior^b^	7	Oogenesis	14
Protein Complex Assembly	9	Polysaccharide Metabolic Process	6
Dorsal Closure	7	Neurological System Process (Sensory Perception)^b^	13
Catabolic Process (Alcohol, Glucose, Carbohydrate)	17	Chromosome Organization and Biogenesis	9
Developmental Process	98	Cell-Cell Signaling (Synaptic Transmission)^b^	5
Cofactor Metabolic Process	9	Cytoskeleton Organization and Biogenesis (Actin Filament-Based Process)	9
Ion Transport	17	Nervous System Development (Axonogenesis, Generation of Neurons)^b^	10
Reproduction (Gamete Production)	24	Biological Regulation (Regulation of Cellular Metabolic Process, Regulation of Gene Expression)	40
Localization	89	Cell Communication	28
Cell Communication (Signal Transduction)	74	Multicellular Organismal Process (Developmental Process/Neuronal Process) b	63
Post-Embryonic Development (Sensory Organ Development)^b^	25		
Nervous System Development^b^	21		
Protein Modification Process (Phosphorylation)	26		
Microtubule-Based Process	6		
Cell Communication (Gene Expression)	74		
Imaginal Disc Development	15		
Chromosome Organization and Biogenesis	7		
Cell Cycle	8		
Cellular Localization (Protein Localization)	17		

a Biological processes. Gene ontology analysis of misregulated genes (p≤0.05) shows their linkage with general and diverse biological processes.

b Neuronal linked biological processes.

c Muscle linked biological process.

**Figure 5:**
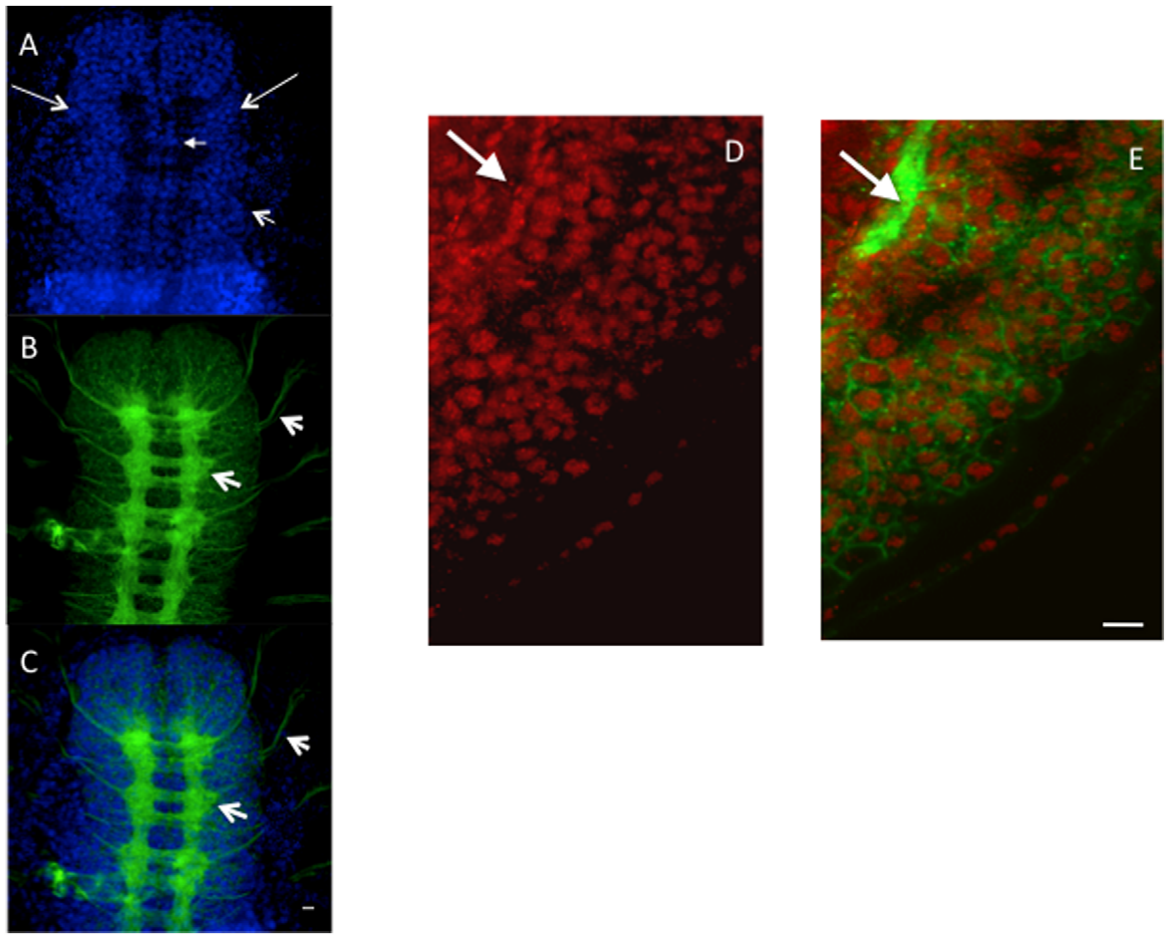
Tip60 localization in the nervous system of *Drosophila* embryos. Confocal microscopic dorsal view of a w^1118^ wild-type embryo (stage 15) double labeled with Tip60 antibody (blue) and horseradish peroxidase (HRP) (green) that labels the cell membrane of all neurons. (**A**) Tip60 Ab staining in the anterior portion of the embryo. dTip60 is present in the central nervous system, and is localized within the anterior brain neuroblast population known as the neuropil (all anterior blue cells on right and left side of embryo, 2 long line arrows), median cells of the CNS (small thin arrow) and possibly within the ganglion cells (short line arrow) [34]. (**B**) HRP labeled anterior portion of the nervous system that labels the cell membrane of all neurons. (**C**) dTip60 and HRP confocal images merged image. dTip60 appears to be localized within the neuronal CNS cells (no co-localization of dTip60 with HRP) and is absent in the segmental and intersegmental axons (thick arrows; **B**, **C**) as visualized by confocal imaging of merged HRP and dTip60 immunostaining at 60× magnification. (**D**) Stage 15 embryo double labeled with dTip60 Ab (red) and HRP Ab (green). Lateral view of the ventral nerve cord showing presence of dTip60 in the CNS and (**E**) dTip60 and HRP merged image showing dTip60 (red) is preferentially localized within the neuronal cells of the fly embryo CNS as indicated by no co-localization with the HRP (green) labeled neuronal membranes. Scale bar: 10 um.

Our microarray data supports a role for Tip60 in neuronal linked processes. This finding prompted us to ask whether dTip60 was produced in the nervous system of the developing fly. Examination of the spatial distribution of the dTip60 protein in the *Drosophila* embryo at high resolution using immunohistochemistry with antibodies specific for the dTip60 protein revealed that despite its low global protein expression pattern during late embryonic stages, dTip60 is produced robustly in the central nervous system, and is preferentially localized within the anterior brain neuroblast population known as the neuropil, CNS mid-line cells and possibly within the ganglion cells (Figure 5A and B) [34], but absent in the growing intersegmental axons (Figure 5C–E). Consistent with our finding that dTi60 HAT activity regulates an array of nervous system specific genes, we found that dTip60 appears to be localized to the nucleus as it is found inside the developing CNS cells (Figure 5 C–E). Robust dTip60 production was also observed within adult fly brains (data not shown). To directly test whether dTip60 HAT activity is essential for neuronal development and function, we targeted dTip60^E431Q^ specifically to the nervous system using three nervous system GAL4 drivers: *elav-* GAL4 (Bloomington, no. 458; [35], [36], [37], and GAL4 179y (Bloomington, no. 3733; [38], [39] which produce robust levels of GAL4 throughout the entire nervous system (pan neuronal expression patterns), and 60IIA-GAL4 (Bloomington, no. 7029) shown by us and others [6], [40], [41] to direct GAL4 specifically to the brain and CNS. For a control, w^1118^ flies were crossed to these three neuronal GAL4 driver lines and showed normal development and no observable phenotypes. However, induction of dTIP60^E431Q^ using insertion lines B and C caused a reduction in viability to 0% for all three GAL4 drivers while line A reduced viability to approximately 25% for elav-GAL4 (Table 3), 30% for 179y-GAL4, and 40% for 60IIa-GAL4 crosses (data not shown). Such variability between independent lines may be due to the varying levels of mutant dTip60 protein production caused by transgene position effects and may indicate that a certain threshold level of dTip60 is required for normal nervous system function. Similar results were obtained for dTip60 knockdown using our three independent dTip60^RNAi^ knockdown fly lines crossed to the elav-GAL4 driver (Table S3) and 179y-GAL4 and 60IIa-GAL4 drivers (data not shown). Taken together, our data suggest that dTIP60 controls neuronal specific gene expression profiles that are required for appropriate development and function of the nervous system.

**Table 3:** Expression of dTip60^E431Q^ using the pan-neuronal elav-GAL4 driver leads to lethality.

Fly Lines x elav-GAL4		
	Number of Surviving Flies^b^	
Test Cross Fly Lines^a^	GAL4-(♂)	GAL4+(♀)
w^1118^	100±4	76±18
dTIP60^E431Q^A	86±2	22±4*
dTIP60^E431Q^B	91±5	0±0*
dTIP60^E431^C	88±6	0±0*
dTIP60^WT^A	67±3	69±4
dTIP60^WT^B	43±10	54±6
dTIP60^WT^C	52±12	64±10

*p≤0.05.

a Test Cross Fly Lines. Five male flies homozygous for either dTip60^E431Q^ or dTip60^WT^ P-element insertions or control w^1118^ were mated to 10 female virgin flies homozygous for the pan-neuronal elav-GAL4 driver located on chromosome X. Adult progeny were counted over an eight day period and the total male (GAL4−) and female (GAL4+) numbers were scored.

b Number of Surviving Flies. Both dTIP60^E431Q^ lines showed significant lethality, with 25% survival for line A and 0% survival for line B and C. Both dTIP60^WT^ lines showed no observable phenotypic effects. The results are reported as mean ± SD, (n = 3).

## Discussion

To create a suitable *in vivo* model to exclusively explore the role of Tip60 HAT activity in developmental gene control during multicellular development, we created transgenic flies producing a dominant negative HAT defective version of Tip60 by introducing the amino acid substitution E431Q into its conserved catalytic HAT domain. Although the corresponding mutation in the Tip60 yeast homolog EsaI (E338Q) was shown to retain proper folding, and display a dominant negative effect on yeast cell growth by specifically disrupting EsaI HAT activity *via* putative disruption of the proton extraction capability of the enzyme [28], it was unknown whether the mutant dTip60 protein would display similar dominant negative effects in the multicellular model system of *Drosophila*. Here, we show that production of dTip60^E431Q^ in flies causes both a reduction in endogenous acetylated H4 histones *in vivo* and a dominant negative lethal effect with increasing severity correlating with higher levels of mutant dTip60^E431Q^. Based on these results, we speculate that the mutant dTip60^E431Q^ protein may produce its dominant negative effect in the fly by outcompeting endogenous wild-type dTip60 for recruitment to chromatin when over-expressed, thus titrating out endogenous histone H4 chromatin acetylation, with resultant deleterious effects on gene expression. Taken together, our findings support a critical role for dTip60 catalytic HAT residue E431 in the acetylation of histone H4 *in vivo* and show that dTip60 HAT activity is essential for multicellular development and are consistent with prior studies demonstrating and essential role for Tip60 in fly [6] and mouse [42] development. As Tip60 plays an important role in regulating apoptosis [43], [44] and double stranded break repair [45], [46], [47], [48], lethality may result, at least in part, by defects in multiple cell division pathways. These findings support that our system is a novel and valuable model for investigating the effects of epigenetic modifications, especially of the Tip60 HAT enzyme, on the developmental processes *in vivo*.

Our microarray analysis of the genome-wide gene changes that result in flies in response to HAT mutant dTip60^E431Q^ production revealed that the majority of misregulated genes clustered into 17 significantly enriched groups, with 8 of these groups each linked to metabolic processes including amino acid, carbohydrate, lipid, glycoprotein and fatty acid metabolism. The significant enrichment of these Tip60 HAT affected metabolic genes supports a central role for Tip60 HAT function in general cellular metabolism. Our findings are consistent with previous studies directly linking Tip60 in the epigenetic based transcriptional control of the central metabolic regulator LRP1 [49], a lipoprotein receptor essential for lipid and cholesterol metabolism. Tip60 also serves as a co-activator for the regulation of transcription factor peroxisome proliferator-activated receptor γ (PPARγ) target genes that play key roles in the regulation of lipid and glucose metabolism [50]. Importantly, a recent elegant study using protein acetylation microarray analysis in yeast demonstrated that the NuA4 complex (yeast homolog of the human Tip60 complex), and specifically EsaI (yeast Tip60 homolog), controls the activity of the central glucose metabolism regulator phosphoenolpyruvate carboxykinase (Pck1p) *via* its direct acetylation [51]. Based on this finding, we speculate that the Tip60 HAT metabolic associated direct and indirect target genes we identified may not only be controlled epigenetically by Tip60 HAT action, but may also represent indirect targets of central metabolic regulator proteins that are directly controlled *via* their acetylation by Tip60. Of note, the majority of misregulated genes we identified in response to dTip60 HAT depletion were upregulated (Figure 3), supporting a critical role for Tip60 HAT activity in the repression of target genes, possibly by the direct recruitment and interaction of Tip60 with transcriptional silencers and/or histone deacetylases that are dependant upon Tip60 acetylation for complex formation or *via* specific Tip60 chromatin acetylation marks that promote recruitment of such silencers to these genes, or by reorganization of chromatin into a repressive environment [52], [53]. Involvement of Tip60 in transcriptional repression is not unprecedented, with a previous study supporting a critical role for Tip60 in epigenetically repressing a large number of developmental genes essential for embryonic stem cell (ESC) differentiation [25]. Additionally, expression of the yeast homolog of our dTip60^E431Q^, HAT-defective dominant negative Esa1^E338Q^ leads to transcriptional silencing of ribosomal DNA (rDNA) in yeast *via* reorganization of nucleolar chromatin structure [7]. Moreover, a recent microarray analysis of RNAi induced Tip60 knockdown in *Drosophila* embryonic S2 cell culture also revealed a significant portion of genes that were upregulated in response to loss of dTip60 activity [54]. Comparison of our data with this previous study using DAVID analysis using our data analysis methods (see methods) revealed 11 identical clusters between the two sets of upregulated gene data that included immune responses, transmembrane transport, cell adhesion, protein modification, morphogenesis and importantly, diverse metabolic processes and nervous system development. However, unlike the above mentioned study [54], we did not identify strong enrichment of genes with chromatin-related annotations among the “repressed” genes we identified and only approximately 28% of the misregulated genes overlap. Such differences may be due to the different starting material (embryonic *Drosophila* cell culture versus whole larval preparation) and knockdown systems (RNAi versus dTip60^E431Q^) used in the Schirling et. al. and Lorbeck et. al. studies, respectively. These differences are important as they suggest that dTip60 may regulate different sets of genes as development proceeds.

Epigenetic regulation has been postulated to provide a coordinated system of regulating gene expression at each stage of neurogenesis, thus promoting brain and CNS development, neural plasticity, learning, and memory [55], [56], [57], [58], [59], [60], [61], [62]. The identification of a number of neurological disorders that result from HAT misregulation underscores a crucial role for acetylation in proper CNS development [63]. For example, missense mutations in the CBP and p300 genes or loss of a CBP allele cause Rubinstein-Taybi syndrome (RTS) [64], [65], [66], [67], a human disease that displays complex phenotypic abnormalities including short stature, learning difficulties, and neoplasia. Moreover, memory loss associated with RTS is specifically due to lack of CBP HAT activity which can be reversed by treatment with specific histone deacetylase inhibitors (HDACs) [65], [66], [68], indicative of a critical role for appropriate histone acetylation in long-term potentiation, learning, and memory. Consistent with these studies, here we provide evidence supporting a role for Tip60 HAT activity in regulating neuronal gene expression profiles required for nervous system function. We show that dTip60 protein is robustly produced in the embryonic nervous system, is localized in the nuclei of brain and CNS cells, and that depletion of Tip60 HAT activity in these tissues results in fly lethality. Importantly, our gene ontology (GO) analysis shows good correlation with these dTip60 protein localization studies in that a substantial number of dTip60 HAT dependent target genes are enriched for neuronal related processes, with 17 clusters linked to diverse nervous system processes and one cluster linked to muscle development. Intriguingly, these were the only tissue-specific related processes identified in our microarray analysis, although we are aware that some cell-specific processes may have been diluted out due to the mixed whole larvae sample preparations used for analysis. A role for dTip60 in neuronal specific function is not unprecedented, with a previous study identifying the dTIP60 gene through its accession number as a potential novel neural precursor gene in a *Drosophila* differential embryonic head cDNA screen [69], although its identity at the time remained uncharacterized. Moreover, preferential expression of TIP60 in the mouse brain has been reported [18] and a recent study reported Bap55 as a chromatin remodeling factor that functions through the TIP60 complex to regulate olfactory projection neuron dendrite targeting in *Drosophila* [70]. Taken together, our results demonstrate yet another example of the importance of HAT function during neurogenesis, and add dTip60 to the growing list of HAT chromatin regulators critical for nervous system function.

Recent studies support an emerging hypothesis that inappropriate changes of specific acetylation marks in chromatin in the adult brain lead to gene misregulation that drives cognitive decline and specifically, memory impairment [71], [72]. These studies demonstrate that in learning assays, aged mice show a specific deregulation of histone H4 lysine 12 (H4K12) acetylation that corresponds with the misregulation of hippocampal gene expression profiles associated with learning and memory [71]. Importantly, these effects can be reversed by restoring physiological levels of H4K12 acetylation. Thus, it is postulated that as individuals age, the accumulation of inappropriate changes in H4K12 acetylation, as well as additional acetylation and methylation marks, lead to altered transcription of neurogenic genes with subsequent negative consequences on cognitive function [72]. Although the HAT activity of CBP has been implicated in learning and memory linked gene regulation, additional specific HATs important in these processes remain to be identified. Here, we show that Tip60 protein is produced robustly in specific cells of the brain and CNS (Figure 5), and that Tip60 HAT activity is essential for appropriate levels of endogenous histone H4 acetylation, *in vivo* (Figure 3). Moreover, we show that Tip60 is essential for brain and CNS development (Table 3), and intriguingly, is linked to the regulation of certain neuronal genes associated with various forms of behavior, learning, memory and synaptic function processes. Based on these results, it is tempting to speculate that Tip60 HAT activity may be involved in marking CNS chromatin important for learning and memory-linked gene regulation. Consistent with this concept, Tip60 HAT activity has been implicated in the age-related neurodegenerative disorder Alzheimer's disease (AD) *via* its HAT dependent complex formation with the C-terminal fragment of the amyloid precursor protein (AICD-APP) and linker protein Fe65 [73], [74], [75]. Recruitment of this complex is critical for the epigenetic regulation of certain genes linked to AD progression [74], [76], [77]. Future investigation into the molecular mechanisms underlying Tip60 HAT function in specific neuronal processes in the fly, particularly those associated with learning and memory, should enhance our understanding into the link between acetylation, cognitive aging and age-related neurodegenerative disorders.

## Materials and Methods

### Construct generation

#### Mutagenesis

BLAST searches were carried out using the BLAST algorithm at NCBI with sequences corresponding to dTIP60 (NP_572151.1) and ESA1 (NP_014887.1) to identify dTIP60 amino acid position E431 corresponding to the catalytic core E338 residue in yeast ESA1. Codon substitution GAG to CAG was incorporated into dTIP60 cDNA construct [6] using the PCR based Quickchange Site Directed Mutagenesis Kit (Quigen, Almeda, CA, USA). Forward and reverse PCR primers are 5′GGCAAGACGGGATCGCCG**CAG**AAACCATTGTCTGATC3′ and 5′GATCAGACAATGGTTT**CTG**CGGCGATCCCGTCTTGCC3′ respectively, with the mutated amino acid code shown in bold. PCR reactions for the mutant strand synthesis reaction contained 25 ng of dTip60 template DNA, 125 ng each of forward and reverse primer, and *PfuTurbo* DNA polymerase (Stratagene, La Jolla, CA, USA). The cycling parameters were 15 cycles of 95° for 30 seconds, 55° for 1 minute, and 68° for 12 minutes using Mastercycler (Epindorf, Madison, WI, USA). Non-mutated methylated parental strands were digested using DpnI (New England Biolabs, Inc., Ipswich, MA, USA) and nicks were repaired upon transformation into DH5α super competent cells (Invitrogen Corporation, Carlsbad, CA, USA). The entire dTip60^E431Q^ construct was sequenced by the University of Pennsylvania DNA Core Sequencing Facility (Philadelphia, PA, USA) for verification of final construct.

#### Cloning procedures

The dTIP60^E431Q^ construct was subcloned into the pUAST GAL4 inducible expression vector [27] as follows. The full open reading frame (ORF) of dTIP60 containing the E431Q mutation was amplified by PCR using forward primer 5′-CGG C*GA ATT C* GC CAA CAT GAA AAT TAA CCA CAA ATA TGA G-3′ containing an EcoRI site (italics), a KOZAC sequence (underlined), and a sequence corresponding to the first eight codons of dTIP60, and reverse primer 5′-GGT T*GG TAC C* TC ATC ATC ATT TGG AGC GCT TGG ACC AGT C-3′ containing a Bam HI restriction site (italics), two in-frame stop codons (underlined), and the last eight codons of dTIP60 [6]. PCR reactions were carried out with the Expand High Fidelity PCR system (Roche, Nutley, NJ, USA) using 400 nM of each forward and reverse primer and cycling parameters of 30 cycles of 95°C for 2 min, 55°C for 1 min, and 72°C for 4 min, using a Mastercycler (Eppendorf, Madison, WI, USA). After digestion and ligation into the pUAST vector, the entire dTIP60^E431Q^ insert was sequenced by the University of Pennsylvania DNA Core Sequencing Facility (Philadelphia, PA, USA) for verification of final construct.

### Drosophila stocks

P-element germline transformations were performed by Rainbow Transgene (Newbury Park, CA, USA) to generate multiple independent fly lines containing either the dTip60^E431Q^ or dTip60^WT^ transgenes. GAL4 drivers used in this study were as follows: Ubiquitous drivers were Act5c-GAL4 (Bloomington Stock Center, no. 4414; Y. Hiromi, 1985) and GAL4 line 337 [29]. The neuronal drivers used were pan-neuronal drivers elav-GAL4 (Bloomington Stock Center, no. 8760 or 8765, and 179y-GAL4 (Bloomington Stock Center, no. 3733; [38], [39] and CNS and brain specific 60IIa-GAL4 (Bloomington Stock Center, no. 7029; [6], [40], [41]. All crosses were performed in triplicate using ten newly eclosed virgin females and five males in narrow plastic vials (VWR International, West Chester, PA, USA) with yeasted *Drosophila* media (Applied Scientific Jazz Mix *Drosophila* Food, Thermo Fisher Scientific, Waltham, MA, USA) at 25°C.

### Quantitative Real Time RT-PCR

Total RNA was isolated from staged three day old larvae using Trizol (Invitrogen Corporation, Carlsbad, CA, USA) and treated twice with Dnase II (Ambion, Austin, TX) to remove DNA. Complementary DNA (cDNA) was synthesized from 1 ug total RNA and oligo-dT primers using Superscript II Reverse Transcriptase (Invitrogen Corporation, Carlsbad, CA,USA). Real-time quantitative PCR was performed on an ABI 7500 Real Time PCR System (Applied Biosystems, Foster City, CA, USA) using the Power SYBR Green PCR master mix (Applied Bioystems, Poster City, CA, USA). All PCR reactions were carried out in triplicate in 20 ul reaction volumes containing 1 ng cDNA template and 500 nm each of forward and reverse primer designed using the NCBI/Primer-BLAST which uses Primer 3 and BLAST (www.ncbi.nlm.nih.gov/tools/primer-blast/). Forward and reverse primer sets designed to amplify a 97 bp non-conserved region of dTIP60 were 5′GACGGCTCACAAACAGGC3′ and 5′GGTGTTGCGGTGATGTAGG, respectively. Forward and reverse primers designed to amplify a 105 bp region within the 5′UTR region of dTIP60 were 5′CAGTTGTGGTCACAATTACCC3′ and 5′GTGCGCAGAAAGTTATACAGC3′, respectively. PCR was carried out by 40 cycles at 95°C for 45 sec, 55°C for 45 sec, and 72°C for 1 min with plate readings recorded after each cycle. Threshold cycle (Ct) values were obtained, and the comparative Ct method was used as previously described [78] to calculate the fold difference in transcript level of the sample relative to the control. RP49 which encodes the ribosomal protein L32 was used as an internal standard and reference gene using forward and reverse primer pairs 5′CTGCTCATGCAGAACCGCGT3′ and 5′GGACCGACAGCTGCTTGGCG3′, respectively.

### Microarray

#### Probe preparation and microarray experiment

Two samples of thirty-five staged three day old whole larvae progeny were collected from each respective genotypic cross. Total RNA was extracted from each of these sample pools using Trizol (Invitrogen Corporation, Carlsbad, CA, USA) and treated two times with Dnase II (Ambion, Austin, TX) to remove genomic DNA. Each of these sample pools was used to probe a separate microarray chip, and thus the mean expression values for each of the three genotypic groups analyzed is the average of 70 individual larvae. Complementary DNA (cDNA) was synthesized from 1 ug total RNA using oligo-dT primers and Superscript II Reverse Transcriptase (Invitrogen Corporation, Carlsbad, CA, USA). RNA quality check, target labeling, GeneChip hybridization, and oligonucleotide microarray scanning were carried out at Seqwright (Houston, TX, USA) on the GeneChip *Drosophila* 2.0 Array (Affymetrix, Santa Clara, CA) following a standard Affymetrix protocol.

#### Data Analysis

Affymetrix GeneChip Operating Software (GCOS) was used to quantitate each GeneChip to produce a .CEL file. GeneChIP.CEL files were loaded into DNA-Chip Analyzer (dCHIP) [79] (http://www.dchip.org) for normalization to reduce technical variation between chips, standardization to reduce variance of expression level estimates by accounting for probe differences, and analysis using model-based expression indexes (MBEI). Correlation matrix analysis was also performed using dCHIP, validating significant consistency of the microarray data for each of the three genotypes analyzed. The dCHIP t-test function was used to identify genes whose expression differed significantly (p<0.05) and these genes were then filtered to select for those that showed a twofold or greater change and a 90% confidence bound of fold change. Correlation coefficients calculated in dCHIP showed significant agreement between duplicate samples for all three genotypes analyzed. Genes were annotated and biological processes were analyzed using the Database for Annotation, Visualization, and Integrated Discovery (DAVID) (http://www.david.abcc.ncifcrf.gov) [32], [33]. Significance of overrepresentation of Gene Ontology (GO) terms was determined p<0.05. A number of significantly misregulated gene targets identified by microarray analysis were validated using qPCR of selected genes using aliquots of the same sample pools prepared for probe labeling and primer sets designed by NCBI/Primer-BLAST (www.ncbi.nlm.nih.gov/tools/primer-blast/). Primers are available upon request.

### Immunohisochemistry and confocal microscopy

w^1118^ embryos were collected and staged over 15–17 hours, dechorionated, fixed in 4% paraformaldehyde and devitellanized. The fixed embryos were incubated in primary antibody overnight. Stained embryos were washed with 1XPBS-T (0.1% Tween) six times over a three hour period (30 minutes each) and were next incubated with the appropriate secondary antibodies for 3 hours at room temperature. Embryos were washed six time over a 3 hour period (30 minutes each). The embryos were next mounted onto slides and imaged using the FV1000 Laser Scanning Confocal Microscope. The following antibodies were used for staining: rabbit polyclonal anti-Tip60 (1∶100) (custom made Tip60 peptide antibody generated by Strategic Diagnostics; www.sdix.com ), FITC tagged goat anti-horse radish peroxidase (HRP) (1∶25; Jackson Immunoresearch). Anti-rabbit fluorescent antibody Alexa Flour 647 for Tip60 visualization was obtained from Invitrogen.

### Western Blot

Histones were isolated from 50 staged second instar larvae using a modified acid extraction protocol [80]. Larvae were homogenized in hypotonic buffer containing 10 mM Tris–HCl (pH 7.4), 3 mM MgCl_2_, 10 mM NaCl in the presence of protease inhibitors and 10 mM Sodium butyrate. After 10 min on ice, 10 µl of 10% TritonX-100 was added and the solution was briefly vortexed. Following 15 s centrifugation, the nuclei were resuspended in 40 µl of nuclear wash buffer (15 mM Tris–HCl (pH 7.4), 60 mM KCl and 15 mM NaCl). Histones were extracted in the presence of 0.4 M HCl for 1 h on ice with regular shaking. After centrifugation, acid-soluble proteins were precipitated with Trichloroacetic acid, washed twice with acetone, air-dried, and resuspended in 50 µl of SDS sample buffer. Equal amounts of protein as quantitated by using a protein assay kit (Thermo Scientific) were loaded onto a 18% SDS PAGE gel (29∶1 acrylamide/bisacrylamide). Protein samples were denatured at 95°C for 15 min prior to loading. The fractionated proteins were electro-blotted onto nitrocellulose membrane (Biorad). The membrane was blocked with 3% BSA for 2 h at room temperature and then incubated overnight at 4°C with an antibody (Ab Serotec, AHP418 ) that recognizes four acetylated lysine residues (K5, K8, K12 and K16) of histone H4. The membrane was washed three times with 0.1% TBST (50 mM Tris-Hcl (pH 7.4), 150 mM NaCl, 0.3% Tween 20) and incubated with secondary antibody for 1 h at room temperature. The membrane was washed three times with 0.1% TBST. Western detection was done using chemiluminiscence (ECL kit, Thermo Scientific). Signals were quantitated using a Fluorchem imager (Alpha Innotech). To ensure signals were in the linear range, Alpha Ease FC software (Alpha Innotech, San Leandro, CA) was used according to the manufacturer's instructions to select exposure times such that there was no saturation detected. Additionally, western blot analysis using the anti-pan H4 Ab was carried out on serial dilutions of purified histones in the range used for this experiment to further ensure that the ECL detection limits used for this analysis were well within the linear range. Western analysis was repeated three separate times with three independent tissue extractions.

### Accession Numbers

The data discussed in this publication have been deposited in NCBI's Gene Expression Omnibus (GEO, http://www.ncbi.nlm.nih.gov/geo/) and are accessible through GEO Series accession number GSE13878.

## Supporting Information

Table S1 ^a^ Test Cross Fly Lines. Ten flies homozygous for the dTIP60^E431Q^ P-element insertion or control w^1118^ were mated to seven flies homozygous for the actin GAL4 driver line *Act5c-GAL4*: P{*Act5c*-GAL4}/*CyO,y^+^*). For independently derived fly lines dTip60^E431Q^ A through D, the P-element insertions are located on chromosome 3. ^b^ Control Cross Fly Lines. Ten flies homozygous for the dTIP60^WT^ P-element insertion were mated to seven flies homozygous for the actin GAL4 driver line *Act5c-GAL4*: P{*Act5c*-GAL4}/*CyO,y^+^*). For independently derived fly lines dTIP60^WT^ A through D, the P-element insertions are located on chromosome 2. ^c^ Rescue Cross Fly Lines. Four independent rescue lines were generated, each homozygous for dTip60^WT^ (line A or B) on the second chromosome and dTip60^E431Q^ (line A or B) on the third chromosome, as described in Table 1. Ten homozygous flies for each of the independent rescue lines were crossed to seven flies homozygous for the ubiquitous 337-GAL4 driver. ^d^ Number of Surviving Flies. Adult progeny were counted over an eight day period and scored for either GAL4^+^(*y;Cy^+^*) or GAL4^−^(*y^+^;Cy*) phenotypes. All four independent dTip60^E431Q^ fly lines reduced viability to 0%, whereas the dTip60^WT^ and w^1118^ control lines showed no observable phenotype. All four rescue lines showed significant rescue of the observed lethal phenotype. The UAS titration control dTip60^E431^/UAS-GFP (described in Table 1) showed no significant rescue, indicating that rescue is dependent upon additional dTip60^WT^ levels, and not potential GAL4 titration due to the additional UAS construct. The results are reported as mean ± SD (n = 3); * p≤0.05.
(DOCX)

Table S2 ^a^ Probe set. ^b^ Listed is the gene name or CG accession number if the gene is uncharacterized.
(DOCX)

Table S3 ^a^ Test Cross Fly Lines. Five male control w^1118^ flies, or five male flies containing dTip60^RNAi^ P-element insertions, were mated to ten female virgin flies homozygous for the pan-neuronal elav- GAL4 driver located on chromosome X. The P-element insertion is located on the X chromosome for Dmel\TIP60/RNAi line A, and the second chromosome for lines B and C. ^b^ Number of Surviving Flies. Adult progeny were counted over an eight day period and the total number of male (GAL4−) and female (GAL4+) flies were scored. dTIP60^RNAi^ lines A–C showed significant lethality, with 0% survival for line A, 27% survival for line B, and 0% survival for line C. Control w^1118^ showed no observable phenotypic effects. The results are reported as mean ± SD, (n = 3).
(DOCX)
