# Supplementary material for: Microarray Analysis Uncovers a Role for Tip60 in Nervous System Function and General Metabolism
Source: PLoS One. 2011 Apr 11;6(4):e18412. doi: 10.1371/journal.pone.0018412 (PMC3073973; doi:10.1371/journal.pone.0018412)
Supplement: Table S1 — a Test Cross Fly Lines. Ten flies homozygous for the dTIP60E431Q P-element insertion or control w1118 were mated to seven flies homozygous for the actin GAL4 driver line Act5c-GAL4: P{Act5c-GAL4}/CyO,y+). For independently derived fly lines dTip60E431Q A through D, the P-element insertions are located on chromosome 3. b Control Cross Fly Lines. Ten flies homozygous for the dTIP60WT P-element insertion were mated to seven flies homozygous for the actin GAL4 driver line Act5c-GAL4: P{Act5c-GAL4}/CyO,y+). For independently derived fly lines dTIP60WT A through D, the P-element insertions are located on chromosome 2. c Rescue Cross Fly Lines. Four independent rescue lines were generated, each homozygous for dTip60WT (line A or B) on the second chromosome and dTip60E431Q (line A or B) on the third chromosome, as described in Table 1. Ten homozygous flies for each of the independent rescue lines were crossed to seven flies homozygous for the ubiquitous 337-GAL4 driver. d Number of Surviving Flies. Adult progeny were counted over an eight day period and scored for either GAL4+(y;Cy+) or GAL4−(y+;Cy) phenotypes. All four independent dTip60E431Q fly lines reduced viability to 0%, whereas the dTip60WT and w1118 control lines showed no observable phenotype. All four rescue lines showed significant rescue of the observed lethal phenotype. The UAS titration control dTip60E431/UAS-GFP (described in Table 1) showed no significant rescue, indicating that rescue is dependent upon additional dTip60WT levels, and not potential GAL4 titration due to the additional UAS construct. The results are reported as mean ± SD (n = 3); * p≤0.05. (DOCX) [file pone.0018412.s001.docx]

**Table S1.** **Ubiquitous expression of dTIP60 using actin driver Act5c produces a dominant negative lethal effect that can be rescued by an additional copy of wild-type dTIP60.**

| **Fly Lines x Act5c** |  | |  | |
| --- | --- | --- | --- | --- |
| **Test Cross Fly Lines^a^** | **Number of Surviving Flies^d^**  **GAL4-(*y*+; *Cy*) GAL4+(*y*; *Cy*+)** | | | |
| w^1118^ | 47±10 | 47±2 | |  |
| dTIP60^E431Q^A | 57±28 | 0±0* | |  |
| dTIP60^E431Q^B | 72±41 | 0±0* | |  |
| dTIP60^E431Q^C | 45±8 | 0±0* | |  |
| dTIP60^E431Q^D | 77±31 | 0±0* | |  |
| **Control Cross Fly Lines^b^** |  |  | |  |
| dTIP60^WT^A | 55±8 | 48±6 | |  |
| dTIP60^WT^B | 62±12 | 62±3 | |  |
| dTIP60^WT^C | 57±8 | 48±8 | |  |
| dTIP60^WT^D | 52±4 | 62±11 | |  |
| **Rescue Cross Fly Lines^c^** |  |  | |  |
| dTIP60^Rescue^A | 53±10 | 68±17 | |  |
| dTIP60^Rescue^B | 65±2 | 69±8 | |  |
| dTIP60^Rescue^C | 58±15 | 63±6 | |  |
| dTIP60^Rescue^D  dTip60 ^UAS titration control^ | 49±5  62±8 | 38±11  0±0* | |  |

*p≤0.05
