# Supplementary material for: Microarray Analysis Uncovers a Role for Tip60 in Nervous System Function and General Metabolism
Source: PLoS One. 2011 Apr 11;6(4):e18412. doi: 10.1371/journal.pone.0018412 (PMC3073973; doi:10.1371/journal.pone.0018412)
Supplement: Table S2 — a Probe set. b Listed is the gene name or CG accession number if the gene is uncharacterized. (DOCX) [file pone.0018412.s002.docx]

**Table S2. Genes that are misregulated in response to both TIP60^E431Q^ and TIP60^WT^.**

| **Probe Set^a^** | **Gene Name^b^** | **Proposed Function** |
| --- | --- | --- |
| 1635824_at | Tip60 | Histone Acetyltransferase |
| 1624926_at | CG13557 | unknown |
| 1624543_s_at | - | unknown |
| 1631056_at | CG17742 | unknown |
| 1633819_at | - | unknown |
| 1628911_at | - | unknown |
| 1634098_at | - | unknown |
| 1641383_s_at | - | Protein Folding |
| 1641162_at | CG31859 | unknown |
| 1626697_at | - | unknown |
| 1632849_at | No optic lobe | Phosphate transport; neuroblast proliferation |
| 1630310_at | CG32751 | Nitrogen compound metabolic process |
| 1624897_at | Lethal(1)G0237 | Chromosome segregation; mitotic metaphase plate congression |
| 1631414_at | CG11313 | proteolysis |
| 1636402_at | CG10081 | proteolysis |
| 1624805_at | CG31041 | unknown |
| 1629934_at | CG14219 | unknown |
| 1627551_s_at | Attacin///attacin | Immune response; antibacterial humoral response |
| 1640884_at | CG15784 | unknown |
| 1632504_at | CG17637 | Transport; tetracycline transport; response to antibiotic |
| 1637886_at | CG10339 | Transport |
| 1623215_at | - | unknown |
