# Supplementary material for: Microarray Analysis Uncovers a Role for Tip60 in Nervous System Function and General Metabolism
Source: PLoS One. 2011 Apr 11;6(4):e18412. doi: 10.1371/journal.pone.0018412 (PMC3073973; doi:10.1371/journal.pone.0018412)
Supplement: Table S3 — a Test Cross Fly Lines. Five male control w1118 flies, or five male flies containing dTip60RNAi P-element insertions, were mated to ten female virgin flies homozygous for the pan-neuronal elav- GAL4 driver located on chromosome X. The P-element insertion is located on the X chromosome for Dmel\TIP60/RNAi line A, and the second chromosome for lines B and C. b Number of Surviving Flies. Adult progeny were counted over an eight day period and the total number of male (GAL4−) and female (GAL4+) flies were scored. dTIP60RNAi lines A–C showed significant lethality, with 0% survival for line A, 27% survival for line B, and 0% survival for line C. Control w1118 showed no observable phenotypic effects. The results are reported as mean ± SD, (n = 3). (DOCX) [file pone.0018412.s003.docx]

**Table S3.** **Expression of dTIP60^RNAi^ using the pan-neuronal elav-GAL4 driver leads to lethality.**

| **Fly Lines x elav-GAL4** |  |  |
| --- | --- | --- |
| **Test Cross Fly Lines^a^** | **Number of Surviving Flies^b^**  **GAL4-(♂) GAL4+(♀)** | |
| w^1118^ | 77±31 | 58±9 |
| dTIP60^RNAi^A | 55±8 | 0±0* |
| dTIP60^RNAi^B | 62±12 | 17±3* |
| dTIP60^RNAi^C | 65±8 | 0±0* |

*p≤0.05
